# Utility of the deep learning technique for the diagnosis of orbital invasion on CT in patients with a nasal or sinonasal tumor

**DOI:** 10.1186/s40644-022-00492-0

**Published:** 2022-09-22

**Authors:** Junichi Nakagawa, Noriyuki Fujima, Kenji Hirata, Minghui Tang, Satonori Tsuneta, Jun Suzuki, Taisuke Harada, Yohei Ikebe, Akihiro Homma, Satoshi Kano, Kazuyuki Minowa, Kohsuke Kudo

**Affiliations:** 1 Department of Diagnostic Imaging, Graduate School of Medicine, Hokkaido University, N15 W7, Kita-Ku, Sapporo, Hokkaido 060-8638 Japan; 2 Department of Diagnostic and Interventional Radiology, Hokkaido University Hospital, N14 W5, Kita-Ku, Sapporo, Hokkaido 060-8648 Japan; 3 Department of Nuclear Medicine, Hokkaido University Hospital, N14 W5, Kita-Ku, Sapporo, Hokkaido 060-8648 Japan; 4 Clinical AI Human Resources Development Program, Faculty of Medicine, Hokkaido University, N15 W7, Kita-Ku, Sapporo, Hokkaido 060-8638 Japan; 5 Department of Radiology, Teine Keijinkai Hospital, 1-40, Maeda 1-12, Teine-ku, Sapporo, Hokkaido 006-8555 Japan; 6 Center for Cause of Death investigation, Faculty of Medicine, Hokkaido University, N15 W7, Kita-Ku, Sapporo, Hokkaido 060-8638 Japan; 7 Department of Otolaryngology-Head and Neck Surgery, Faculty of Medicine and Graduate School of Medicine, Hokkaido University, N15 W7, Kita ku, Sapporo, 060-8638 Japan; 8 Faculty of Dental Medicine, Department of Radiology, Hokkaido University, N13 W7, Kita-ku, Sapporo, Hokkaido 060-8586 Japan; 9 Global Center for Biomedical Science and Engineering, Faculty of Medicine, Hokkaido University, N14 W5, Kita-Ku, Sapporo, Hokkaido 060-8638 Japan

**Keywords:** Head and neck, Nasal or sinonasal tumor, Orbital invasion, Periorbita, Deep learning, Transfer learning

## Abstract

**Background:**

In nasal or sinonasal tumors, orbital invasion beyond periorbita by the tumor is one of the important criteria in the selection of the surgical procedure. We investigated the usefulness of the convolutional neural network (CNN)-based deep learning technique for the diagnosis of orbital invasion, using computed tomography (CT) images.

**Methods:**

A total of 168 lesions with malignant nasal or sinonasal tumors were divided into a training dataset (*n* = 119) and a test dataset (*n* = 49). The final diagnosis (invasion-positive or -negative) was determined by experienced radiologists who carefully reviewed all of the CT images. In a CNN-based deep learning analysis, a slice of the square target region that included the orbital bone wall was extracted and fed into a deep-learning training session to create a diagnostic model using transfer learning with the Visual Geometry Group 16 (VGG16) model. The test dataset was subsequently tested in CNN-based diagnostic models and by two other radiologists who were not specialized in head and neck radiology. At approx. 2 months after the first reading session, two radiologists again reviewed all of the images in the test dataset, referring to the diagnoses provided by the trained CNN-based diagnostic model.

**Results:**

The diagnostic accuracy was 0.92 by the CNN-based diagnostic models, whereas the diagnostic accuracies by the two radiologists at the first reading session were 0.49 and 0.45, respectively. In the second reading session by two radiologists (diagnosing with the assistance by the CNN-based diagnostic model), marked elevations of the diagnostic accuracy were observed (0.94 and 1.00, respectively).

**Conclusion:**

The CNN-based deep learning technique can be a useful support tool in assessing the presence of orbital invasion on CT images, especially for non-specialized radiologists.

## Background

Malignant sinonasal tumors often involve the orbit, and orbital invasion is associated with a significant reduction in survival [1]. Cross-sectional imaging is essential for the staging and preoperative assessment of sinonasal tumors in order to determine resectability [2, 3]. If orbital invasion is suspected, the surgeon and the patient are confronted with the difficult decision of exenteration. The relationship between the tumor and the periorbita is one of the criteria in the determination of whether exenteration is necessary; tumor invasion beyond the periorbita may warrant exenteration, whereas an intact periorbita warrants preservation [1, 4]. Extraocular muscle involvement and orbital fat obliteration were reported to have high positive predictive values of orbital invasion beyond periorbita on computed tomography (CT) and magnetic resonance imaging (MRI) [4]. However, the diagnosis of orbital invasion beyond periorbita requires experience and is difficult even for experienced radiologists, and even more so for radiologists who are not specialists in this area.

The use of artificial intelligence (AI) has continued to increase in various fields as a problem-solving technique that replicates human intelligence through computer and algorithmic technologies [5]. Artificial intelligence using deep learning (DL) techniques, such as a convolutional neural network (CNN), has fostered hopes for and research toward revolutionizing the automated analysis of medical images. Deep learning algorithms have been applied to medical imaging in several clinical settings and have attracted considerable attention, as their use has been demonstrated to perform at least as well as humans in image classification tasks [6, 7]. We speculated that using AI may enable the determination of orbital invasion beyond periorbita on CT and help radiologists evaluate such cases.

We conducted the present study to (1) develop a CNN model that can be used to diagnose the presence of orbital invasion on CT, (2) evaluate the diagnostic performance of the developed CNN model, and (3) assess the effect of its utility on the diagnostic performance of general radiologists who are not specialized in the image reading of the head and neck.

## Methods

This retrospective study was approved by the Institutional Review Board of the Hokkaido University, and the requirement for patients’ written informed consent was waived.

### Study population

**Fig. 1:**
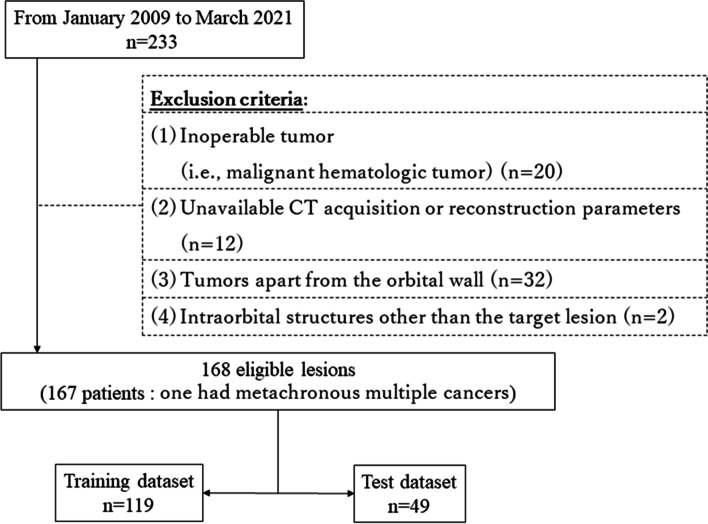
Study population, study flow, and recruitment pathway

Based on the medical records, we selected the cases of 233 patients who were treated at our hospital during the period from January 2009 to March 2021, with the following inclusion criteria: (1) patients with a pathologically confirmed malignant nasal or sinonasal tumor and (2) pretreatment coronal CT images reconstructed with soft tissue kernel including the tumor lesion. Some patients were excluded by the following exclusion criteria: (1) patients with an inoperable tumor regardless of the presence of the orbital invasion (e.g., a malignant hematologic tumor) (*n* = 20), (2) CT acquisition or reconstruction parameters (e.g., slice thickness, matrix size, convolution kernel, etc.) were not available (*n* = 12), (3) patients whose primary tumor was located clearly apart from the orbital wall (*n* = 32), and (4) intraorbital structures which severely affected the imaging findings of the target lesion, such as an artificial eye (*n* = 2). Ultimately, 167 patients and their 168 lesions (one of the patients had metachronous multiple cancers) were considered eligible for this study. We randomly selected 119 lesions as the training dataset to create the diagnostic model, and we used the other 49 lesions as the test dataset to evaluate the performance of the established model, each approx. 7:3 ratio (Fig. 1).

### CT images

CT images of the total 168 lesions were obtained by various scanners from four vendors. Of the 168 lesions, 150 were post-contrast enhanced images and the other 18 lesions were non-contrast enhanced images. We used the coronal reconstructed CT images for the evaluation. Other image parameters were as follows. Slice thickness: 1–3 mm, matrix size: around 512 × 512, reconstruction kernel: soft tissue.

### Determination of the final diagnosis

Two board-certified radiologists with 6 and 15 years of experience in head and neck radiology determined whether the quality of the CT images was appropriate for interpretation. Subsequently, all of the coronal CT images were divided into orbital invasion beyond periorbita-positive or -negative (hereinafter, referred to as “invasion-positive or -negative”) groups by these two radiologists in consensus, using a Digital Imaging and Communication in Medicine (DICOM) viewer (XTREK, J-MAC SYSTEM, Tokyo). Imaging findings of the bone destruction of orbital wall, the presence of irregularity between the tumor margin and orbital components, extraocular muscle involvement by the tumor, and the orbital fat obliteration around the tumor were carefully assessed, and invasion-positive or -negative status was determined in each image by taking all of imaging findings into consideration. After this image assessment, a total of 81 cases were diagnosed as invasion-positive and the other 87 cases were diagnosed as invasion-negative. Approximately 9 months after the above-mentioned consensus reading, the two board-certified radiologists re-evaluated all cases individually to divide them into invasion-positive and negative cases in order to determine the inter-observer agreement in the case-based invasion-positive and -negative decisions they had made.

After the case-based evaluation for the division of invasion-positive and -negative cases, for the preparation of the training dataset, we further performed slice-based evaluation to divide the invasion-positive and -negative slices within each positive case; this procedure was conducted in the training dataset only. In each orbital invasion-positive case, all slices in the range of evaluation (i.e., the range from the nasolacrimal duct orifice to the tip of the middle cranial fossa) were assessed and divided into invasion-positive slices and -negative slices by the abovementioned two radiologists. In contrast, all of the CT images in the orbital invasion-negative cases were assigned as invasion-negative slices.

To assess the variability of consensus reading by board certified radiologists in determining the invasion-positive and -negative status, other two board-certified radiologists with 7 and 12 years of experience evaluated all cases by consensus to divide into invasion-positive and -negative, as an additional consensus reading session.

### Image analysis

#### Image selection and post-processing for the deep learning analysis

**Fig. 2:**
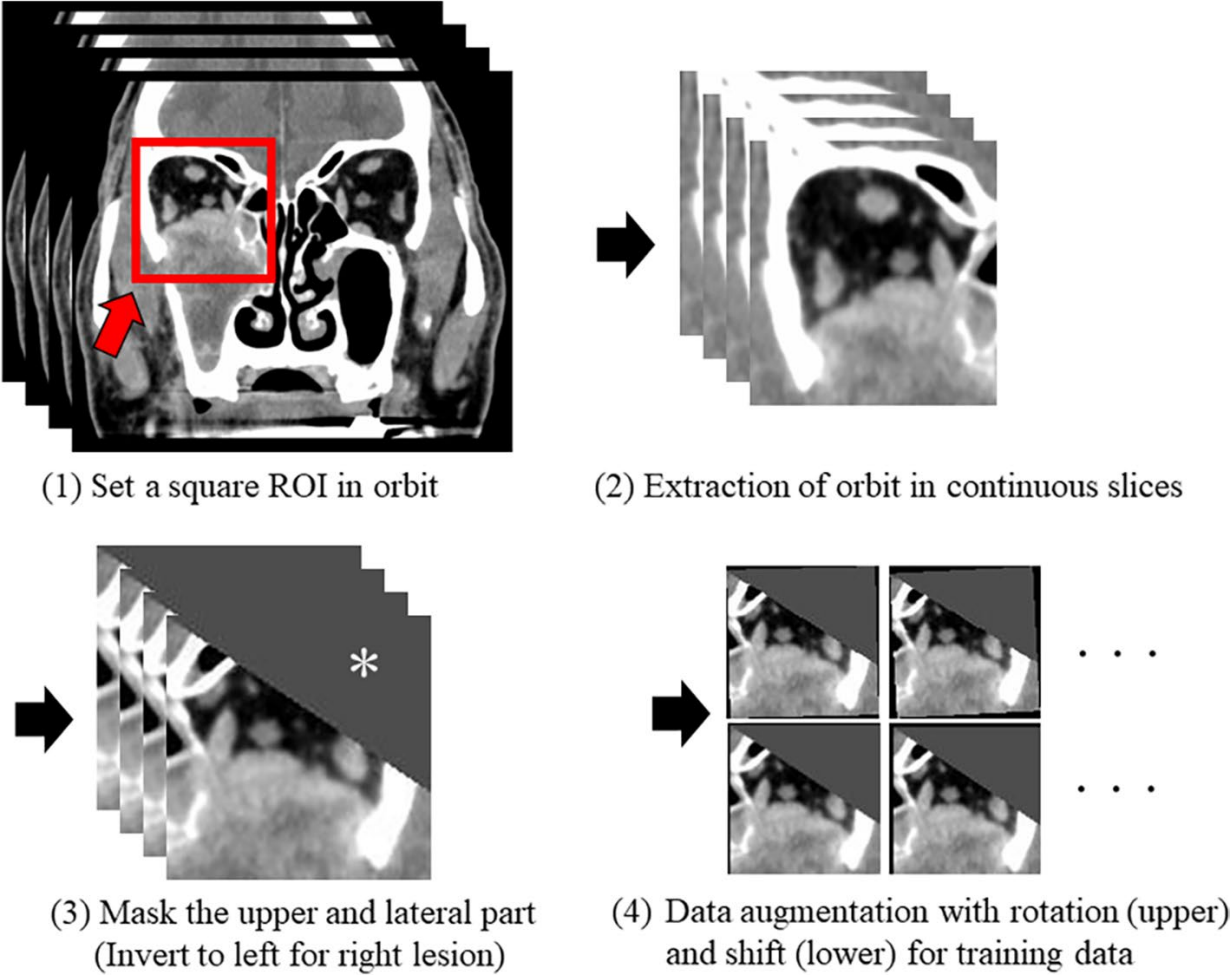
Image preprocessing on CT images for the deep learning analysis. First, a square region of interest (ROI) was manually placed to fully include the orbital wall on coronal CT images (red arrow). Next, segmented images by the ROI were extracted as continuous slices including the tumor. Then, right-side lesion images were inverted to the left side (flipped horizontally) and all images were aligned, as the lesion is shown at the left side. Thereafter, the top and outer areas in the image were masked (white asterisk). Finally, these processed images were fed into the data augmentation process with image rotation and/or a shift for training data

We randomly selected a training data set to create a diagnostic model and a test data set to evaluate the performance of the established model (see below Results). First, image segmentation was performed on all coronal CT images. We manually drew the square regions of interest (ROIs) to encompass the orbital bone wall with an ROI size of approx. 12 cm^2^. If the target tumor was located around the midline and contacted the bilateral orbit, the ROIs were separately drawn for both right and left sides. We used CT images in the range from the nasolacrimal duct orifice to the tip of the middle cranial fossa for the ROI placement. However, specific CT image slices in which the primary tumor was observed to be far from the orbital wall were excluded for further analysis. The segmented images of the right orbit were then horizontally flipped and all aligned, and they were observed in the same manner as the left orbit. A triangular mask was applied on the upper and lateral parts of the orbit. The CT window of all images was adjusted to the window level 60/window width 300 Hounsfield units (HU). Finally, each processed image was output as a Joint Photographic Experts Group (JPEG) document. The image processing steps are illustrated in Fig. 2.

For the preparation of the invasion-positive group in the training dataset, we included only the specific slices that were judged as invasion-positive in the slice-based evaluation (see above: Determination of the final diagnosis); this dataset consisted of 408 images from 56 lesions. In contrast, we included slices on which the tumor lesion was in contact with the orbital wall but had not invaded periorbita in the invasion-negative group; this dataset consisted of 635 images from 63 lesions. Before the training session, data augmentation was performed to improve the robustness of the model, with random rotation and vertical and/or horizontal shifting for each image; a total of 10 additional images were generated for each image.

In the test dataset, all images in all lesions (both invasion-positive and -negative) were used; this consisted of 25 invasion-positive lesions (191 invasion-positive images and 123 invasion-negative images; every lesion included at least one invasion-positive image) and 24 invasion-negative lesions (326 invasion-negative images). The test dataset was evaluated without a data augmentation procedure.

### Deep learning analysis

We classified the invasion-positive or -negative status of the coronal CT images by using transfer learning from a pre-trained CNN algorithm devoted to image classification. The original model used in this work was the Visual Geometry Group 16 (VGG16) model, developed at Oxford University in 2015, which had been trained and evaluated on the ImageNet collection (http://image-net.org/index) [8]. The VGG16 model is composed of 16 layers with a combination of five convolutional blocks (13 convolutional layers) and three fully connected layers, finishing with a dense layer that operates the final classification through 1000 different categories proposed by the ImageNet dataset [8]. Thanks to its simplicity, the VGG16 model is well adapted for transfer learning for a small dataset [9]. For the model’s training, the last fully connected layer was trained, whereas the parameters of other prior layers were fixed to the original weights of the VGG16 model. This allowed us to keep the more generic features of the VGG16 model and adapt the model to the CT images through a limited number of trainable parameters.

For the training session, the stochastic gradient descent with momentum (sgdm) optimizer was used. Hyperparameters were set to 15 epochs, a mini-batch size of 32, and the learning rate 1.0 × 10^− 5^. In the transfer learning, 30% of the training data was used as internal validation during the training. The VGG16 model is able to convert the input image into a probability regarding the category in which it would belong, and in our present study the VGG16 model outputted a binary classification of invasion-positive or -negative status on the test dataset. The model was established using an Ubuntu 18.04 long-term support (LTS)-based server with a Core i9 10980XE 18core/36thread 3.0-GHz central processing unit (CPU), four NVIDIA Quadro RTX8000 graphics processing unit (GPU) cards, and 128-GB (16GB × 8) DDR4–2933 quad-channel memory for training and validation. The time required for an epoch was approx. 14 min, and approx. 13 epochs were enough to reach the final score evaluated on the test dataset. All image analyses were performed using MATLAB (R2021a, MathWorks, Natick, MA, USA) and Metavol software (https://www.metavol.org) [10].

### Visual evaluation by radiologists

Two general radiologists with 6 and 3 years of experience who were not specialists in the interpretation of head and neck images reviewed all of the images in the test dataset (49 lesions; 25 invasion-positive/24 negative) and independently determined whether the tumor lesion was invasion-positive or -negative. They referred to all of the slices with a complete field of view (not segmented images) for the evaluation. For patients with lesions bordering the bilateral orbits, they evaluated the right and left sides separately to determine the presence of invasion. At approx. 2 months after the first reading session, the same two general radiologists reviewed all of the images in the test dataset again to determine the invasion-positive or -negative status, referring to the patient-based diagnoses provided by the CNN model developed with VGG-16 described above.

### Statistical analyses

The distribution of patient characteristics between the training and test cohorts were compared using the χ2-test for categorical variables and the Mann-Whitney U-test for continuous variables.

We used the kappa coefficient to evaluate the inter-observer agreement regarding the case-based invasion-positive and -negative decisions made by the two board-certified radiologists with extensive head-and-neck imaging experience. The kappa coefficient was also used to assess the agreement between one of the board-certified radiologists and the result of the consensus reading, and between the other board-certified radiologist and the result of the consensus reading. Kappa values < 0.40 were interpreted as poor agreement, 0.41–0.57 as fair agreement, 0.58–0.74 as good agreement, and > 0.75 as excellent agreement [11].

In addition, the agreement between the result of first consensus reading (i.e., the final diagnosis) and that of additional consensus reading by other two board-certified radiologists was assessed using the Kappa coefficient. A receiver operating characteristic (ROC) curve analysis to calculate the area under the curve (AUC) was also performed using the result of the additional consensus reading by setting the result of first consensus reading as the gold standard.

The diagnostic performances for the test dataset obtained with 1) the developed CNN diagnostic model, 2) the general radiologists without the developed CNN diagnostic model’s assistance, and 3) the general radiologists with the developed CNN diagnostic model’s assistance were respectively evaluated. When we evaluated the CNN diagnostic model, we first performed slice-based diagnoses by dividing all slices into those indicating the invasion-positive or -negative status by using the developed CNN model in each patient. Each of the abovementioned slice-based diagnoses was then converted to an individual-based diagnosis by adding up the number of consecutive slices that the CNN model determined to be invasion-positive per one patient. A ROC curve analysis was performed to determine the optimal number of consecutive slices for diagnosing invasion-positive or -negative status as a patient-based diagnosis, using the Youden index. The diagnostic performance was assessed by computing the following performance metrics: the AUC, accuracy, sensitivity, specificity, positive predictive value (PPV), and negative predictive value (NPV). The diagnostic performance achieved by each of the general radiologists without the CNN model’s assistance was compared with that obtained by the CNN model alone and that by the same radiologist with the CNN model’s assistance, respectively. The comparison of the AUC was calculated by the χ^2^-test. Statistical significance was set at *p*-values < 0.05. BellCurve for Excel (Social Survey Research Information Co., Tokyo) was used to perform all statistical analyses.

## Results

### Patient characteristics

Table 1 summarizes the characteristics of the patients in the training, test, and total cohorts. Because one of the 167 patients considered eligible for this study had multiple metachronous carcinomas, each of the patient’s lesions was counted as a separate case. The age of that patient was determined as the time when the malignancy was pathologically diagnosed. In the comparison of patient characteristics between the training and test cohorts, only the patient age was significantly different (*p* = 0.048); no significant between-cohort difference was observed in other characteristics.

**Table 1:** Patients’ characteristics

	Total	Training	Test	*p* value
	(*n* = 168)	(*n* = 119)	(*n* = 49)	
Age, yrs.; median (range)	65 (29–91)	66 (29–86)	49 (30–91)	0.048
Males/females	129/39	88/31	41/8	0.175
Primary site:				0.863
Maxillary sinus	117	84	33	
Nasal cavity	30	22	8	
Ethmoid sinus	17	11	6	
Sphenoid sinus	2	1	1	
Maxillary gingiva	2	1	1	

### Interobserver agreement in the determination of the final diagnosis

The inter-observer agreement between the two board-certified radiologists in their case-based invasion-positive and -negative decisions was excellent (kappa coefficient 0.769, 95%CI: 0.671–0.867). The kappa coefficients for the agreement between the result of the consensus reading and one of the board-certified radiologists was 0.808 (95%CI: 0.720–0.897), and that for the other board-certified radiologist was 0.844 (95%CI: 0.764–0.925).

### Agreement between results of the first and additional consensus reading

The agreement between the result of the first consensus reading (i.e., the final diagnosis) and that of additional consensus reading by other two board-certified radiologists was excellent (kappa coefficient 0.810, 95% CI: 0.722–0.898). In the ROC curve analysis, with setting the result of first consensus reading as a gold standard, the AUC from the results of the additional consensus reading was 0.907 (95% CI: 0.864–0.950).

### Diagnostic performance of the CNN model

To evaluate the performance of the established model, we randomly divided the total of 168 nasal and sinonasal malignant tumors into 119 lesions as the training dataset to create the diagnostic model and 49 lesions as the test dataset (~ 7:3 ratio). One patient in the training dataset and two patients in the test dataset showed an extent to bilateral orbit, and thus the number of cases included 120 lesions in the training dataset (invasion-positive *n* = 57; invasion-negative *n* = 63) and 51 lesions in the test dataset (−positive *n* = 26; −negative *n* = 25).

For the transfer learning with VGG16 using the training dataset, the computed accuracy at the final epoch in the training session (i.e., the accuracy of the internal validation during the training session) was 0.920.

**Fig. 3:**
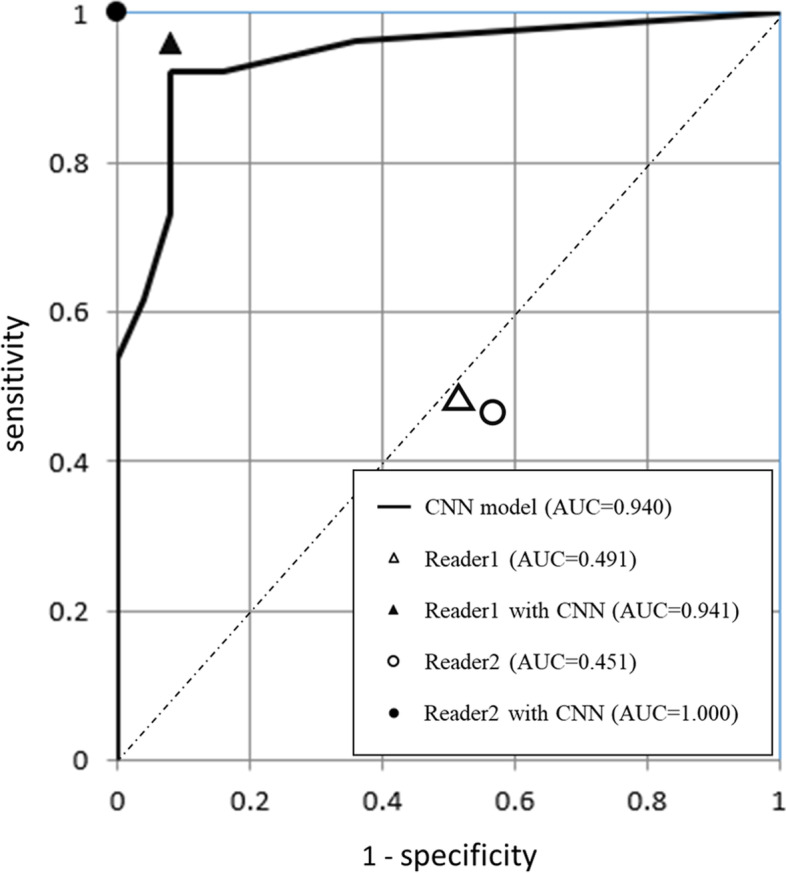
ROC curve analysis. The ROC curve for the CNN-based deep learning models of VGG16 is shown. The point plots of the sensitivity and specificity values of the two non-specialist radiologists with and without the CNN model’s assistance are also shown

Subsequently, the test dataset was imported into the developed CNN model. All images in the test dataset were divided into the invasion-positive or -negative group for each slice. We performed an ROC curve analysis for the differentiation of invasion-positive or -negative lesion status by using the number of consecutive slices that were determined the CNN model to be invasion-positive in each patient of the test dataset. The CNN model-based diagnosis on the test dataset achieved an AUC of 0.940 (95%CI: 0.873–1.000) (Fig. 3). With the use of this cut-off value, when three or more consecutive slices were set as the best cut-off point based on the Youden index, the following values were obtained: 0.922 (95%CI: 0.811–0.978) accuracy, 0.923 (95%CI: 0.749–0.991) sensitivity, 0.920 (95%CI: 0.740–0.990) specificity, 0.923 (95%CI: 0.749–0.991) PPV, and 0.920 (95%CI: 0.740–0.990) NPV (Table 2).

**Table 2:** Diagnostic performances

	CNN model	Reader 1	Reader 1 with CNN	Reader 2	Reader 2 with CNN
AUC	0.940	0.509	0.941	0.549	1.000
Accuracy	0.922	0.490	0.941	0.451	1.000
Sensitivity	0.923	0.462	0.962	0.462	1.000
Specificity	0.920	0.520	0.920	0.440	1.000
PPV	0.923	0.500	0.926	0.462	1.000
NPV	0.920	0.482	0.958	0.440	1.000

*AUC* area under the curve, *CNN* convolutional neural network, *NPV* negative predictive value, *PPV* positive predictive value

### Radiologists’ diagnostic performances

In the blind review of the test set without CNN model assistance, the diagnostic performances of the two non-specialist radiologists were as follows. Reader 1: AUC 0.491 (95%CI: 0.351–0.631), 0.490 accuracy (95%CI: 0.348–0.634), 0.500 (95%CI: 0.291–0.709) sensitivity, 0.481 (95%CI: 0.287–0.681) specificity, 0.462 (95%CI: 0.266–0.666) PPV, and 0.520 (95%CI: 0.313–0.722) NPV; Reader 2: AUC 0.451 (95%CI: 0.311–0.590), 0.451 accuracy (95%CI: 0.311–0.597), 0.462 (95%CI: 0.266–0.666) sensitivity, 0.440 (95%CI: 0.244–0.651) specificity, 0.462 (95%CI: 0.266–0.666) PPV, and 0.440 (95%CI: 0.244–0.651) NPV.

**Fig. 4:**
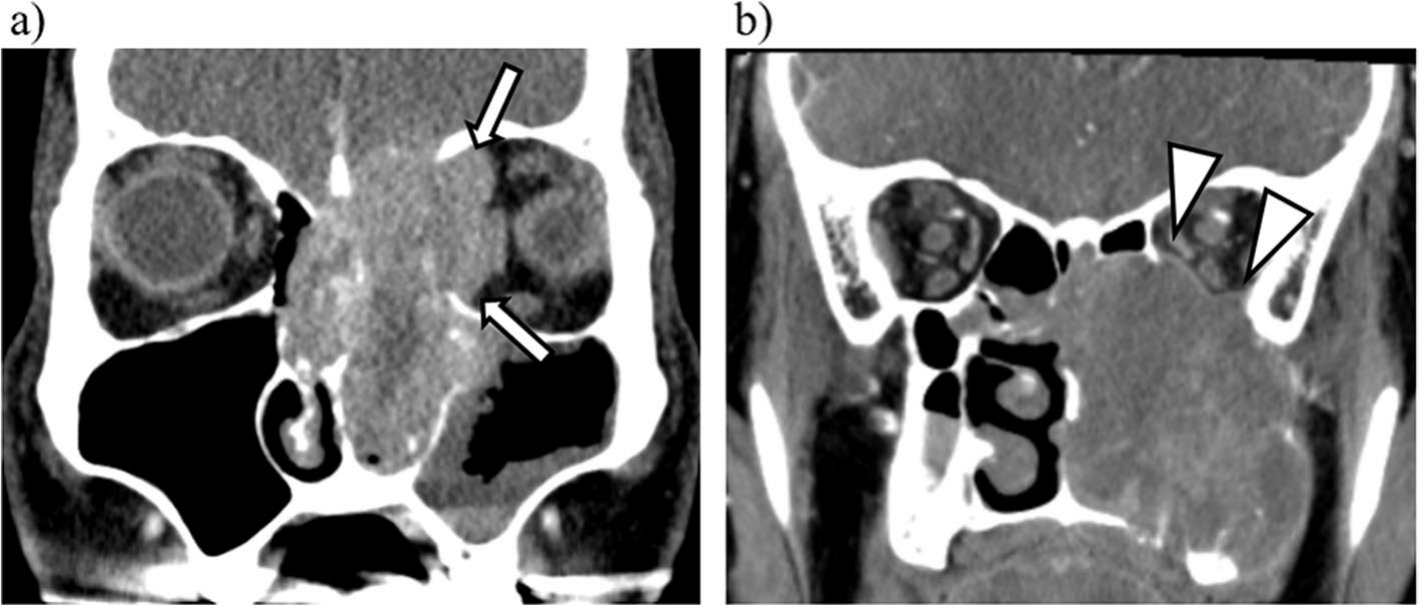
Representative cases diagnosed as false-negative and false-positive with the trained CNN model. **a** A patient with left ethmoid sinus cancer. As the tumor involved the extraocular muscles and obliterated orbital fat with medial orbital wall destruction (white arrow), the final diagnosis by experienced radiologists was determined as invasion-positive. However, the CNN model-based diagnosis was negative (a false negative). **b** A patient with left maxillary sinus cancer. As a thin membrane structure bordering the tumor and the intraorbital fat could be identified (white arrowhead), the final diagnosis by the experienced radiologists was invasion-negative. However, the CNN model-based diagnosis was positive (false positive)

Assisted by the CNN model-based diagnosis, both of these radiologists achieved a higher diagnostic performance as follows. Reader 1: AUC 0.941 (95%CI: 0.875–1.007, 0.941 (95%CI: 0.838–0.988) accuracy, 0.962 (95%CI: 0.757–0.991) sensitivity, 0.920 (95%CI: 0.789–0.999) specificity, 0.926 (95%CI: 0.804–0.999) PPV, and 0.958 (95%CI: 0.740–0.990) NPV; Reader 2: AUC 1.000 (95%CI: 1.000–1.000), 1.000 (95%CI: 1.000–1.000) accuracy, 1.000 (95%CI: 1.000–1.000) sensitivity, 1.000 (95%CI: 1.000–1.000) specificity, 1.000 (95%CI: 1.000–1.000) PPV, and 1.000 (95%CI: 1.000–1.000) NPV. Table 2 summarizes the results of the comparison of the non-specialist radiologists’ performances without and with the CNN model’s assistance. The ROC curve obtained from the CNN model in the test dataset and the point plot of the sensitivity and specificity values obtained from the two radiologists’ visual evaluation in the test dataset analysis with and without the CNN model assistance are depicted in Fig. 3. The AUC of the CNN model was significantly higher than those for both radiologists (readers 1 and 2) (*p* < 0.001, respectively). In addition, the AUC obtained by both radiologists with the assistance of the CNN model was significantly higher than the AUC obtained without the assistance (*p* < 0.001). Representative false-positive and false-negative cases with the CNN-based diagnoses are presented in Fig. 4.

## Discussion

The incidence of orbital invasion by malignancies in the nasal or sinonasal cavity varies with the site of origin, histology, and aggressiveness of the tumor. Invasion of the orbital bone wall occurs in 66–82% of ethmoidal malignancies [12] and in 60–80% of maxillary sinus malignancies [13]. The periorbita is the periosteal lining of the internal orbit and covers the four orbital walls from the anterior aperture of the orbital cavity back to the conical apex with the optic canal and the superior orbital fissure [14]. The periorbita has been considered an effective barrier to tumor extension into the orbit [4]. The invasion of orbital contents through the periorbita is one of the general indications for orbital clearance, and such invasion heralds a poorer prognosis [1, 15]. Displacement of the periorbita by a tumor is one of the most accurate radiologic signs of orbital invasion seen on CT, showing an NPV of 86% and a PPV of 75% [4, 16].

In the present study, the use of the DL technique to diagnosis the orbital invasion beyond periorbita in CT was successful. We speculated that the DL technique would successfully train the CNN-based diagnostic model to identify the above-mentioned radiologic signs of orbital invasion observed on CT. To the best of our knowledge, no prior study has assessed the application of the DL technique for the imaging diagnoses of orbital invasion by head and neck malignancies. Investigations of the usefulness of DL approaches to diagnose the presence of local invasion by malignant tumors in CT images include examinations of visceral pleural invasion in early-stage lung cancer [17], muscular invasion in bladder cancer [18], and extranodal extension in lymph node metastases of head and neck squamous cell carcinoma [19]. All of these studies described good diagnostic performance, with some results reported as similar to or better than those obtained by experienced radiologists. Several studies have also indicated the usefulness of the DL technique as a supportive tool that provided improved diagnostic performance with the aid of a DL-based diagnostic model [19–21]. Our present findings demonstrated the same trend of results as those described in these previous studies.

We observed herein that almost all of the cases were diagnosed correctly with the developed CNN model; however, two false-negative and two false-positive cases were detected. Both of the false-negative cases involved the medial wall and not the inferior wall. The lesions with medial wall involvement were mainly from nasal, ethmoidal, and sphenoidal sinus tumors, and the number of tumors at this location was too small compared to the maxillary sinus. The amount and quality of the training with the imaging findings with medial wall involvement for the CNN model’s development might therefore not be sufficient due to the small number of cases.

In contrast, the imaging findings of the two false-positive cases were as follows. Both lesions expanded and projected into the orbit to a certain degree, but the margin was well circumscribed, with thinned orbital bone wall and without irregularities of orbital fat density. Sufficient training of this imaging findings might not be performed in the training session, because the number of cases with such imaging findings in the total cohort was also small.

Our study has the following limitations. The sample size was small due to the single-institutional study design, and the results should thus be treated as preliminary. The CT scanning parameters (e.g., slice thickness, in-plane matrix size) were heterogenous among the cohorts. However, in light of our findings, we believe that using a DL technique could effectively create a diagnostic model even in such a heterogeneous dataset. The inclusion of CT images with various scanning parameters may have allowed us to verify the accuracy of the data in a manner similar to external validation. Most of the cases in the present cohort did not have a surgically confirmed diagnosis of orbital invasion beyond periorbita, and their categorization was based solely on CT imaging findings. This is due to the characteristics of our institution, where the treatment of choice for advanced sinonasal tumors is predominantly chemoradiotherapy, and therefore pathological results are difficult to obtain. Last, only CT features of the orbital invasion beyond periorbita were analyzed; MRI findings were not included. It has been reported that several MRI imaging features are useful for the differentiation between invasion-positive and -negative status [16], but because some of the present patients did not undergo MRI, we did not include MRI findings in this study.

## Conclusion

The CNN model can be useful for the diagnosis of orbital invasion beyond periorbita on CT images. This technique may become a diagnostic support tool, especially for radiologists who are not specialists in head and neck imaging.

## Data Availability

The datasets used and/or analyzed during the current study are available from the corresponding author on reasonable request.

## Abbreviations

AI: Artificial intelligence
AUC: Area under the curve
CNN: Convolutional neural network
CT: Computed tomography
DICOM: Digital Imaging and Communication in Medicine
DL: Deep learning
HU: Hounsfield units
JPEG: Joint Photographic Experts Group
MRI: Magnetic resonance imaging
NPV: Negative predictive value
PPV: Positive predictive value
ROC: Receiver operating characteristic
ROI: Region of interest
sgdm: Stochastic gradient descent with momentum
VGG16: Visual Geometry Group 16
